# ‘Feeling Hot’: Exploring the feasibility of nocturnal erection detection through penile temperature measurements

**DOI:** 10.1002/bco2.372

**Published:** 2024-05-30

**Authors:** Hille J. Torenvlied, Evelien Trip, Wouter Olthuis, Loes I. Segerink, Rob C. M. Pelger, Jack J. H. Beck

**Affiliations:** ^1^ Department of Urology St. Antonius Ziekenhuis Nieuwegein The Netherlands; ^2^ Faculty of Electrical Engineering, Mathematics and Computer Science and BIOS Lab on a Chip Group Universiteit Twente Enschede The Netherlands; ^3^ Department of Urology Leids Universitair Medisch Centrum Leiden The Netherlands

**Keywords:** ambulatory diagnostics, e‐health, erectile dysfunction, nocturnal erections, penile temperature, RigiScan, temperature sensing

## Abstract

**Objectives:**

The observational ‘Feeling Hot’ study aims to evaluate the feasibility of employing overnight penile temperature measurements for the detection of nocturnal erections, thereby contributing to the advancement and modernization of a non‐invasive diagnostic system for erectile dysfunction.

**Subjects/Patients and Methods:**

In this proof‐of‐concept study, 10 healthy men aged 20–25 were recruited, following the methodology outlined in the ‘Staying Hot’ study by Torenvlied et al. Participants underwent ambulatory overnight penile temperature measurements concurrent with RigiScan recordings. Key outcome measures included baseline and peak penile temperatures during RigiScan‐annotated nocturnal erections. Reference measurements of the thigh temperature were also taken to assess nocturnal temperature variations.

**Results:**

Statistically significant penile temperature increases (*p* = 0.008, *n* = 9) were observed during nocturnal erections, with an average elevation of 1.47°C noted during the initial erections. This underscores the practical utility of penile temperature measurements in detecting erection onset. Challenges arose in accurately determining erection duration and subsequent erection onsets due to the persistence of elevated temperatures following initial erections, termed the ‘Staying Hot effect’. Reference thigh temperature measurements aided in addressing this challenge.

**Conclusion:**

Examining overnight penile temperature alongside simultaneous RigiScan recordings has yielded valuable insights into the viability of using the temperature methodology for detecting nocturnal erections. The ‘Feeling Hot’ study findings demonstrate significant penile temperature elevation during nocturnal erections in healthy young men, highlighting the potential of integrating this measurement methodology into the design of a modernized tool for ambulatory erectile dysfunction diagnostics. Further development of an advanced sensor system to comprehensively assess erection duration and quality is essential for enhancing clinical applicability.

## 1 INTRODUCTION

Erectile dysfunction (ED), defined as ‘the inability to attain and maintain an erection sufficient to permit satisfactory sexual performance’, is a common benign condition affecting up to 52% of men aged 40–70 years.^1^ Despite its benign nature, ED significantly impacts the quality of life for both patients and their families.^2^ Accurately distinguishing between organic and psychogenic ED is crucial for effective treatment. Currently, the RigiScan stands as the gold standard diagnostic tool for detecting nocturnal penile erections.^3^, ^4^ Nocturnal erections, occurring automatically and involuntarily during rapid eye movement (REM) sleep, serve as a reliable indicator for normal erectile function.^5^, ^6^ However, the clinical utility of the RigiScan is diminishing due to its cumbersome size, outdated software and suboptimal patient experience, raising concerns about its validity.^4^, ^7^ Consequently, many healthcare institutions resort to invasive alternative diagnostic techniques, emphasizing the urgent need for a modernized system for diagnosing ED.

Non‐invasive measurement of penile rigidity and circumference relies on incorporating a pressure component within the measurement system, which can impact patients' sleep quality and consequently the validity of overnight measurements. Previous research has demonstrated a significant increase in penile temperature during erections in healthy men, both in naked and simulated overnight conditions.^8^, ^9^, ^10^, ^11^, ^12^ This raises the question of whether detecting nocturnal erections through penile temperature measurements, which offer a non‐pressure approach, is feasible. The ‘Feeling Hot’ study aims to address this inquiry by examining penile temperature measurements during RigiScan‐annotated nocturnal erections. A successful feasibility assessment has the potential to pave the way for further validation of a modernized pressure‐free diagnostic system, revolutionizing the differentiation of ED aetiology and facilitating the development of a patient‐friendly tool for ambulatory ED diagnostics.

## 2 SUBJECTS, MATERIALS AND METHODS

### 2.1 Study setting and participants

The observational ‘Feeling Hot’ study, conducted at the Department of Urology, St. Antonius Ziekenhuis, Nieuwegein, the Netherlands, received ethical approval from the medical research ethics committees united on 23 February 2022 (NL79969.100.21, R21.115). Prior to participant enrolment, the proof‐of‐concept study was pre‐registered at ClinicalTrials.gov (NCT05183581).

The findings of the ‘Staying Hot’ study indicate an expected increase in penile temperature during nocturnal erections of 0.67°C (SD 0.41). A power calculation using repeated measures with *α* < 0.05 and *β* = 20% determined a required sample size of *n* = 5. The inclusion criteria of the ‘Feeling Hot’ study closely align with those outlined in the ‘Staying Hot’ study by Torenvlied et al.^12^ Accordingly, we recruited 10 healthy participants aged 20–25, strictly adhering to the exclusion criteria specified in the referenced research. In summary, exclusion criteria included unwillingness to provide informed consent, an International Index of Erectile Function (IIEF)‐5 score below 22 and a medical history involving sickle cell anaemia, atherosclerosis, diabetes or the use of benzodiazepines.^13^ Furthermore, the exclusion criteria for the ‘Feeling Hot’ study were expanded to encompass a medical history of REM‐sleep behaviour disorder, restless legs syndrome, insomnia, sleep apnoea and the use of sleeping pills. Participants were included between April and June 2022.

### 2.2 Materials and study procedure

The temperature measurement system utilized in the ‘Feeling Hot’ study was an expanded version of the temperature sensing system employed in the ‘Staying Hot’ study.^12^ The system comprised three Ohmeda 3P T3312 temperature probes (Shenzhen Medke Technology Co., Shenzhen, Guangdong, China),^14^ connected to a PicoLog 1216 data logger (Pico Technology, Cambridgeshire, UK),^15^ and a stand‐alone Lenovo ThinkBook 13s‐IWL laptop. Positioning the system near the participants' bed facilitated unrestricted movement and reduced entanglement hazards.

Participants were instructed to sleep in cotton underwear and under a blanket, alone and refrain from alcohol consumption, sexual activity or masturbation on the evening of participation. To ensure participant privacy and minimize skin contact duration, participants themselves performed the sensor placement.

The first thermistor temperature sensor was positioned on the dorsal penile skin proximal to the glans. The second temperature sensor, the reference sensor, was placed on the subject's thigh. Both sensors were secured to the skin using Leukosan 12 × 100‐mm strips. The third temperature sensor, placed near the participant's bed, recorded room temperature, ensuring consistency across the study cohort.

Nocturnal erections were annotated using the RigiScan (GOTOP Medical Inc., St. Paul, MN, USA).^3^ Participants followed the standard protocol, placing the two RigiScan loops at the tip and base of the penis and positioning the datalogger in the designated fabric on the subjects' legs. Following sensor placement, participants proceeded to take overnight ambulatory measurements upon entering the bed. The success criteria for the overnight measurements involved the detection of at least one nocturnal erection lasting a minimum of 15 min, as recorded by the RigiScan device.^3^ In the event of missing data, test subjects were excluded.

### 2.3 Data processing and statistical analysis

The temperature data were processed using MATLAB R2019a (The MathWorks, USA), employing the methodologies outlined in the ‘Staying Hot’ study by Torenvlied et al.^12^ RigiScan images were imported into MATLAB, and the ‘ginput’ tool was utilized to extract the precise start and endpoint of nocturnal erections following the standard protocol.^3^ Four outcome variables were defined for temperature data during the annotated erectile periods:

- $T_{\text{peak}}$: the peak temperature during the erectile period;
- $T_{\text{baseline}}$: the minimum temperature in the 15 min preceding the RigiScan‐annotated erectile period;
- ∆T: the temperature difference between T_peak_ and T_baseline_ ( $∆T=T_{\text{peak}}-T_{\text{baseline}})$; and
- erection duration.

To address changes in body temperature resulting from sleeping under blankets and to mitigate temperature fluctuations due to movement, the reference sensor was utilized. The sensor's data were analysed to derive the ‘corrected penile temperature’, defined as the disparity between penile and thigh temperature (T_penis_ − T_thigh_). Values for $T_{\text{peak}}$, $T_{\text{baseline}}$ and ∆T were computed for both the penile temperature data and the ‘corrected penile temperature’.

In addition to calculating the aforementioned variables, a visual assessment of the temperature data was conducted. This involved analysing the curves of both the raw penile temperature and the corrected penile temperature data. Furthermore, slope analysis was performed by determining the derivative (*dT/dt*) of the temperature curve with a time interval (*dt*) set at 20 s. To mitigate short‐term disturbances in penile temperature, temperature values within this 20‐s timeframe were averaged.

The statistical analysis was carried out using IBM SPSS Statistics (Version 25.0, Armonk, NY, USA). Initially, the data underwent normal distribution testing through the Shapiro–Wilk test. Subsequently, the Wilcoxon signed‐rank test was employed to assess significant differences between baseline and peak temperature (Δ*T*). Correlations were computed using Spearman's rho. The threshold for statistical significance was set at *α* < 0.05.

## 3 RESULTS

In this proof‐of‐concept study, a total of 10 overnight measurements were performed. One participant was excluded due to sleep disturbances caused by the RigiScan, preventing sleep. The mean age of participants was 22.1 years (SD 1.97), with a body mass index (*BMI*) of 22.00 kg/m^2^ and a median *IIEF‐5 score* of 24.00 (interquartile range [IQR] 2.00).

Among the remaining nine participants, the RigiScan detected a total of 32 erectile periods, averaging 3.56 periods per subject (SD 1.13) with an average duration of 36.67 min (SD 11.34). The room temperature during the ambulatory measurement averaged 21.79°C (SD 2.69), showing stable overnight curves for each participant. No significant correlation was found between room temperature and baseline temperature (Spearman's rho = 0.633, *p* > 0.05).

**FIGURE 1:**
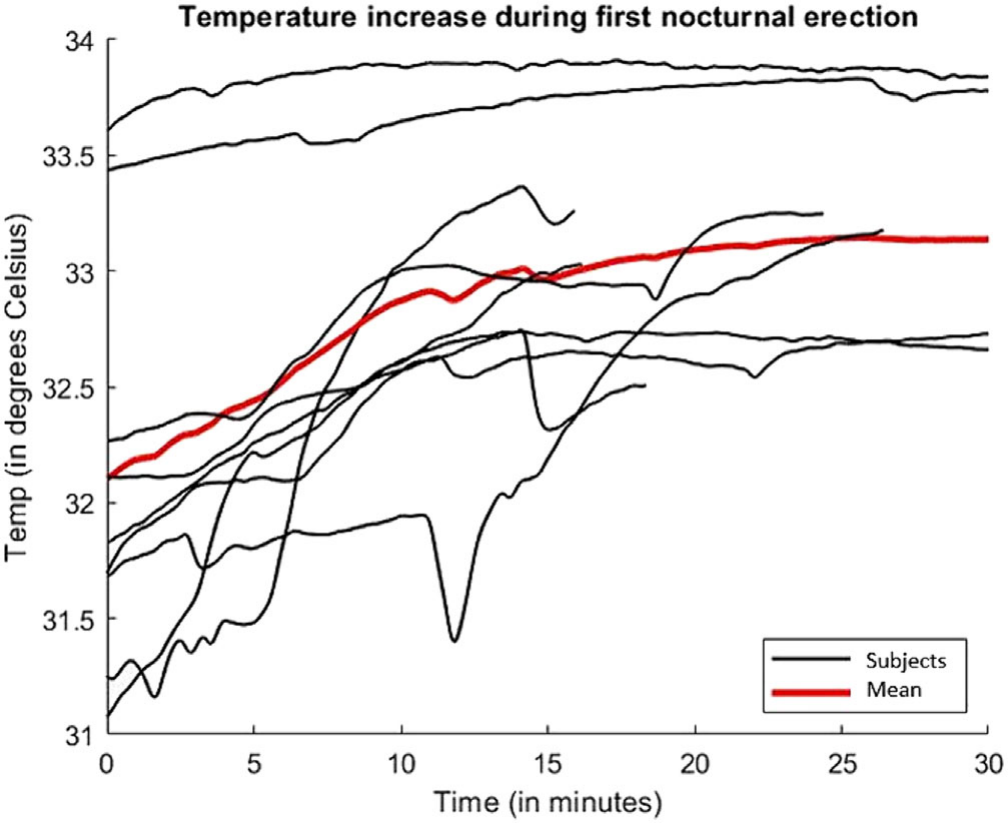
Penile temperature during the initial nocturnal erection of all test subjects. The mean of the data is shown in red.

Table 1 illustrates penile temperature variables for overnight erectile periods. The difference between penile baseline and peak temperature was significant (*p* < 0.01), with an average *∆T* of 0.87°C (SD 0.31). The initial nocturnal erection displayed the highest Δ*T*, with an average increase of 1.47°C (SD 0.87). Figure 1 visually represents the penile temperature increase during the initial erection of all participants. The Δ*T* for the initial erection was significantly higher than subsequent erections due to the significantly lower baseline temperature.

**TABLE 1:** Overview of the outcomes of the penile temperature measurements for the naked and blanket measurements (*n* = 9). Values are given as the mean (SD) or median (IQR), dependent on the distribution of the data. *T*_*baseline*_ is the minimal temperature in the 15 min prior to erection, *T*_*peak*_ is the maximal temperature during erection and Δ*T* is the difference between *T*_*baseline*_ and *T*_*peak*_ during erection.

Variables	All erections	Initial erection
*T*_*baseline*_ (in °C)	32.32 (SD 0.55)	31.73 (SD 1.05)
*T*_*peak*_ (in °C)	33.24 (IQR 0.68)	33.21 (SD 0.44)
*∆T* (in °C)	0.87 (SD 0.31)	1.47 (SD 0.87)

Abbreviation: IQR, interquartile range.

The corrected temperature (T_penis_ − T_thigh_) data mirrored penile temperature results as shown in Table 2. A significant difference existed between baseline and peak temperatures (*p* = 0.008).

**TABLE 2:** Overview of the outcomes of the corrected temperature values. Values are given as the mean (SD). *T*_*baseline*_ is the minimal temperature in the 15 min prior to erection, *T*_*peak*_ is the maximal temperature during erection and Δ*T* is the difference between *T*_*baseline*_ and *T*_*peak*_ during erection.

Variables	All erections	Initial erection
*T*_*baseline*_ (in °C)	−0.17 (SD 0.40)	−0.54 (SD 0.52)
*T*_*peak*_ (in °C)	0.61 (SD 0.38)	0.46 (SD 0.49)
*∆T* (in °C)	0.78 (SD 0.30)	0.99 (SD 0.47)

**FIGURE 2:**
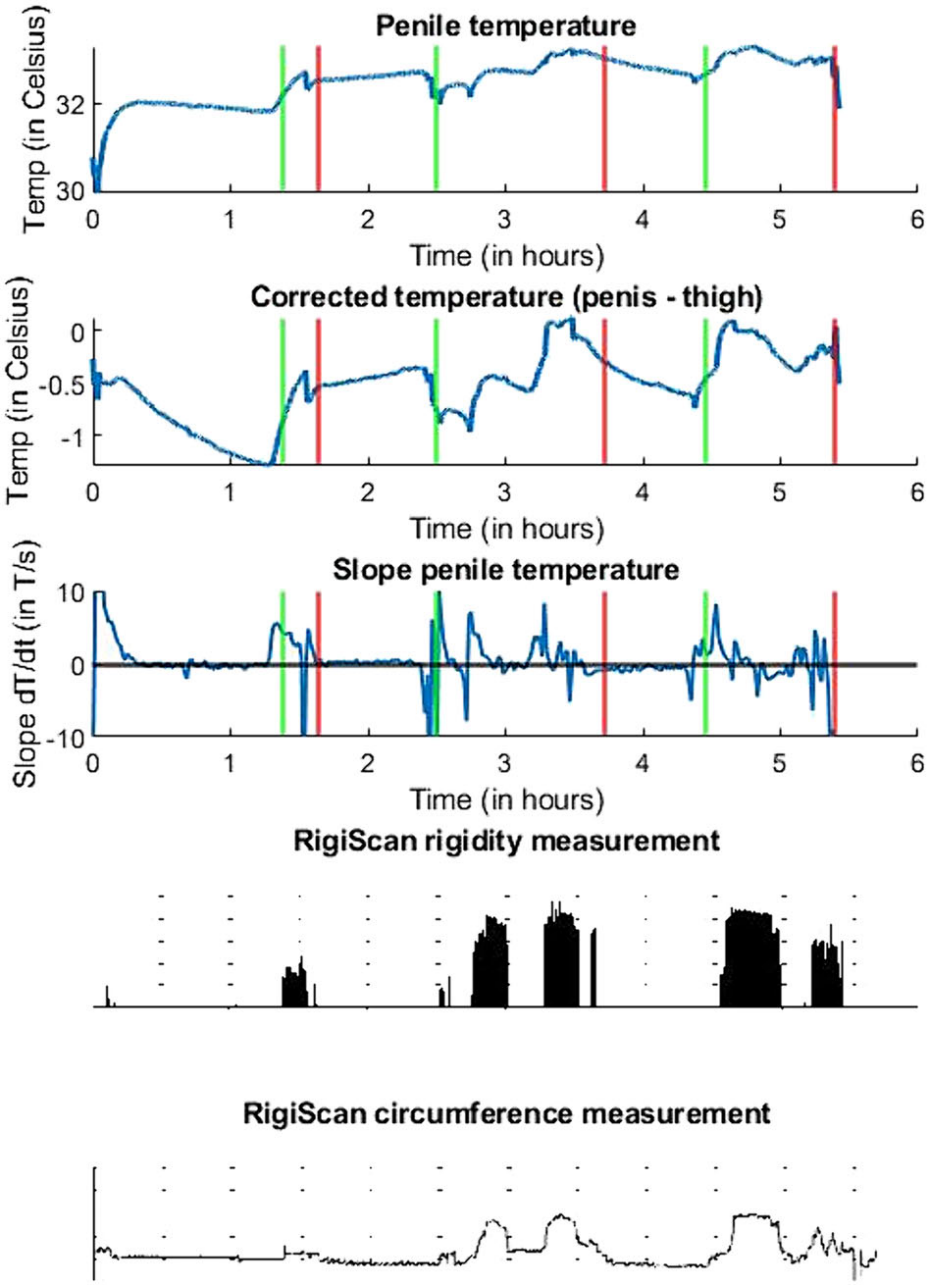
Outcomes of a single overnight measurement of the penile temperature, corrected temperature, penile temperature slope analysis and RigiScan rigidity and circumference measurements. The start and end of the nocturnal erectile phases, according to the RigiScan circumference data, are indicated with green and red lines, respectively.

Figure 2 provides a comprehensive visual of (corrected) penile temperature, slope analysis, and simultaneously measured RigiScan outcomes for a single participant. The initial 15 min demonstrated a penile temperature increase, while the corrected temperature remained stable. The penile temperature stabilized at a value of 32°C, while the corrected temperature decreased until the occurrence of the initial erection. As a result, the subject's thigh temperature increased to 1.5°C above the penile temperature due to environmental conditions (the presence of blankets). The initial nocturnal erection detected by the RigiScan occurred approximately 1 h later. During this period, the penile temperature visibly increased by approximately 1°C.

Figure 2 also shows that following the initial nocturnal erection and during subsequent nocturnal erections, the penile temperature remained at an elevated peak level. The corrected temperature course and slope analysis do correspond to the RigiScan‐annotated rigidity measurements. Clear increases in temperature were observed during the second and third erectile phases in the curve of corrected temperature, indicating an increase in penile temperature relative to the environmental conditions. A slope analysis of the penile temperature provided a more precise measurement of the start and end times of the nocturnal erections, with clear fluctuations in the slope occurring during all three erections. These fluctuations were absent during the non‐erectile phases.

Most overnight curves exhibited patterns similar to Figure 2, with clear detection of an initial penile temperature increase for all participants. Subsequent nocturnal erections were detected in seven subjects through further analysis of corrected temperature and slope curves. However, for two participants, subsequent erections were undetectable in any curves.

**FIGURE 3:**
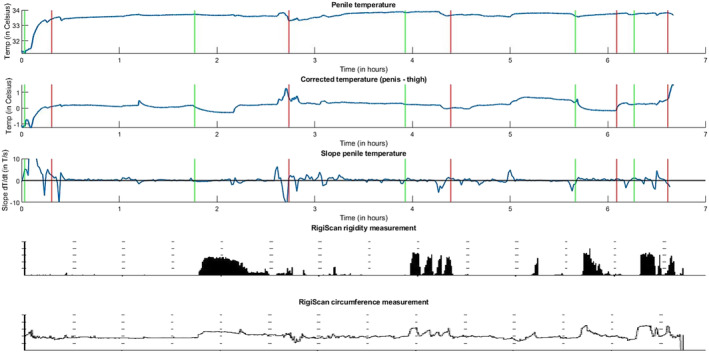
Visualization of the added value of corrected temperature measurements and slope analysis on the detection of (subsequent) nocturnal erections. An overview is given of the penile temperature, corrected temperature, penile temperature slope analysis and RigiScan rigidity and circumference measurements. The start and end of the nocturnal erectile phases, according to the RigiScan circumference data, are indicated with green and red lines, respectively.

Figure 3 illustrates overnight data for one participant with undetectable subsequent nocturnal erections. The corrected temperature showed a decrease during the erections and fluctuations in slope analysis did not align with RigiScan‐annotated rigidity measurements. Consequently, visual detection of subsequent nocturnal erections was not possible for this participant.

Additionally, Figure 3 highlights a distinct phenomenon observed in all test subjects within the initial 15 min of overnight measurements. Following the onset of the temperature sensors and RigiScan, the penile temperature exhibited an approximate increase of 2°C. However, the corrected temperature also displayed an increase of approximately 1°C instead of stabilizing or decreasing. This indicates that the penile temperature experienced a greater increase than what could be attributed to environmental conditions alone. Analysis of the RigiScan data reveals the presence of small rigidity peaks and fluctuations in circumference, suggesting a sexual stimulus. This phenomenon was observed in three subjects.

## 4 DISCUSSION

The ‘Feeling Hot’ study represents the first overnight feasibility assessment of a novel methodology for nocturnal erection detection. The findings indicate a significant increase in penile temperature during these erections in healthy young men, with visible erectile periods evident in overnight temperature curves. These positive outcomes suggest that temperature measurement holds promise as a reliable basis for the advancement of a modernized clinical tool for non‐invasive ED diagnostics.

Preceding the ‘Feeling Hot’ study was the ‘Staying Hot’ study, which explored the impact of overnight conditions on erectile penile temperature in a controlled environment.^12^ The study found an average penile temperature increase of 0.67°C during erection measurements, prompting the question of whether this increase would be adequate for detecting nocturnal erections. In contrast, the current study observed a much higher average penile temperature increase of 1.47°C during the initial erection, affirming the feasibility of detecting nocturnal erections in overnight measurements. Two factors explain the difference in temperature change between the studies. First, the average duration of nocturnal erections in this study was 36.67 min, compared with 7.47 min in the ‘Staying Hot’ study. Second, the baseline temperature was 31.73°C, as opposed to 32.75°C. While the human body temperature remains stable or rises during sexual arousal, it decreases during sleep to conserve energy and facilitate the restoration of fatigued cerebral processes.^16^, ^17^, ^18^

Despite the notable difference in Δ*T* observed between the two studies, the ‘Staying Hot effect’ distinctly emerges in the overnight penile temperature curves. This effect implies that overnight conditions, such as the presence of blankets and underwear, lead to the prolonged maintenance of penile temperature at its peak for over 30 min following the conclusion of an erection.^12^ In the overnight measurements, it was observed that the penile temperature did not revert to baseline values after the initial erection, substantiated by the significant difference in baseline temperature between the initial and subsequent erections.

The influence of the ‘Staying Hot effect’ on the reliability of nocturnal erection detection using the temperature methodology sparks a discussion, particularly in the challenging detection of subsequent nocturnal erections. However, it is crucial to note that the disclosure of the presence of a single erectile period is adequate for diagnosing psychogenic ED, aligning with the primary objective of conducting ambulatory measurements. The initial erection was conspicuously distinguishable in the penile temperature curves of all participants during RigiScan‐annotated nocturnal erections. Consequently, nocturnal erection detection through temperature measurement not only proves feasible but also exhibits diagnostic potential for distinguishing the nature of ED.

The viability of detecting the presence of nocturnal erections using the temperature methodology is evident; however, improving the capability for subsequent erection detection would be beneficial. Figure 2 illustrates that the curves of corrected temperature and slope analysis enabled the visual identification of subsequent nocturnal erections, a capability observed in seven out of the nine participants. It is highly probable that the application of advanced data analysis techniques, such as machine learning techniques, could facilitate the identification of subsequent nocturnal erections in temperature data. Further research is necessary to explore the feasibility and effectiveness of implementing these techniques.

The corrected temperature not only played a crucial role in the detection of subsequent erections but also proved valuable in identifying a sexual stimulus during the initiation of the RigiScan device. It is highly probable that patients undergoing ambulatory overnight measurements for ED diagnostics will have a significantly lower likelihood of experiencing an erection induced by the stimulus of the RigiScan. One limitation of this phenomenon is the inability to differentiate between the temperature increase associated with entering the bed and that caused by the sexual stimulus. This could have potentially led to an overestimation of the actual temperature increase during the initial erection in the participants. It is essential to recognize that the application of the thigh reference temperature for analysing the corrected temperature is necessary in validation studies as long as RigiScan annotation is employed.

The use of the RigiScan as the methodology for annotating erections could be considered another limitation of this study, given concerns raised by previous research about the validity of RigiScan measurements in ED diagnostics.^4^, ^7^ The device can significantly impact the sleep quality of patients, which is critical for obtaining accurate data. However, the data from the healthy volunteers in this study clearly demonstrated phases of penile circumference and rigidity increases, validating the detection of nocturnal erections with the RigiScan. Moreover, the RigiScan remains the gold standard for non‐invasive ambulatory ED diagnostics, as there is currently no validated substitute device available. The experience of a test subject struggling to sleep due to constant sexual stimulation from the RigiScan underscores the importance of developing a modernized device that does not exert pressure or resistance on penile tissue.

While the temperature sensor does not exert pressure on the penile tissue, the results of the ‘Feeling Hot’ study reveal a limitation in the methodology—it only allows for annotating the initiation of an erection. Notably, it cannot discern the commencement of detumescence, making the determination of erection duration impossible. In contrast, the RigiScan not only provides insights into this duration but also assesses erectile quality through penile rigidity measurements. An ideal modernized diagnostic tool should encompass non‐pressure measurement of both erection duration and erectile quality. This could be achieved by integrating multiple physiological principles into a unified (wireless) sensor system, considering factors like penile saturation, pulse or acceleration, especially in line with the goal of avoiding pressure on the penile tissue.^19^

The development of such a sensor system lays the foundation for clinical implementation, which would naturally follow the execution of a validity study conducted in a larger study population. The feasibility of erection detection with the temperature methodology was proven for healthy young men in this study. For validity studies, the system's capability to differentiate between somatic and psychogenic causes in ED patients should be investigated. Understanding the impact of aging or the absence of erections on overnight (temperature) curves is crucial. Additionally, insights into the demographic properties of the ED patient population are necessary to ensure a comprehensive design of future validity and implementation studies.

## 5 CONCLUSION

Examining overnight penile temperature alongside simultaneous RigiScan recordings has provided valuable insights into the potential of utilizing temperature‐based methods for detecting nocturnal erections. The findings of the ‘Feeling Hot’ study reveal a significant penile temperature increase during nocturnal erections in healthy young men, underscoring the feasibility of integrating this approach into a modernized ambulatory diagnostic tool for ED. Nevertheless, further refinement and extension of an advanced sensor system are essential to comprehensively evaluating erection duration and quality, addressing challenges posed to the temperature by the ‘Staying Hot effect’. Despite these challenges, the ‘Feeling Hot’ study represents a significant initial advancement towards modernizing non‐invasive ED diagnostics, paving the way for the reintroduction of patient‐friendly ED diagnostic solutions in clinical settings worldwide.

## AUTHOR CONTRIBUTIONS

All authors have approved the final version of this manuscript. Hille J. Torenvlied conceived and designed the work that led to the submission. Furthermore, she acquired the data, interpreted the results and drafted and revised the manuscript. She is accountable for all aspects of the work, ensuring that questions related to the accuracy or integrity of any part of the work are appropriately investigated and resolved. Evelien Trip collaborated on the interpretation of the results and revised the manuscript. Wouter Olthuis collaborated on the design of the work that led to the submission. He supported the interpretation of the results and revised the manuscript. Loes I. Segerink revised the manuscript. Rob C. M. Pelger revised the manuscript. Jack J. H. Beck supported the design of the work that led to the submission. Furthermore, he collaborated on the interpretation of the results and revised the manuscript.

## CONFLICT OF INTEREST STATEMENT

There are no conflicts of interest associated with this study. No financial benefits or support were received from the distributors of the components of the penile temperature measurement system. There is no (desired) patent holding or stock ownership that could cause a conflict of interest.
